# Perception of risk and communication among conventional and complementary health care providers involving cancer patients’ use of complementary therapies: a literature review

**DOI:** 10.1186/s12906-016-1326-3

**Published:** 2016-09-08

**Authors:** Trine Stub, Sara A. Quandt, Thomas A. Arcury, Joanne C. Sandberg, Agnete E. Kristoffersen, Frauke Musial, Anita Salamonsen

**Affiliations:** 1 Department of Epidemiology and Prevention, Wake Forest School of Medicine, Division of Public Health Sciences, Winston-Salem, NC 27157 USA; 2 Department of Family and Community Medicine, Wake Forest School of Medicine, Winston-Salem, NC 27157 USA; 3 Department of Community Medicine, The National Research Center in Complementary and Alternative Medicine (NAFKAM), UiT The Arctic University of Norway, Tromsø, Norway; 4 Present address: Department of Community Medicine, The National Research Center in Complementary and Alternative Medicine (NAFKAM), UiT The Arctic University of Norway, Tromsø, Norway

**Keywords:** Communication between health care providers, Provider-patient communication, Risk, Patient safety, Complementary therapy, Complementary and Alternative Medicine, Oncology, Cancer care

## Abstract

**Background:**

Communication between different health care providers (conventional and complementary) and cancer patients about their use of complementary therapies affects the health and safety of the patients. The aim of this study was to examine the qualitative research literature on the perception of and communication about the risk of complementary therapies between different health care providers and cancer patients.

**Methods:**

Systematic searches in six medical databases covering literature from 2000 to 2015 were performed. The studies were accessed according to the level of evidence and summarized into different risk situations. Qualitative content analysis was used to analyze the text data, and the codes were defined before and during the data analysis.

**Results:**

Twenty-nine papers were included in the primary analysis and five main themes were identified and discussed. The main risk situations identified were 1. Differences in treatment concepts and philosophical values among complementary and conventional health care providers. 2. Adverse effects from complementary products and herbs due to their contamination/toxicity and interactions with conventional cancer treatment. 3. Health care physicians and oncologists find it difficult to recommend many complementary modalities due to the lack of scientific evidence for their effect. 4. Lack of knowledge and information about complementary and conventional cancer treatments among different health care providers.

**Conclusion:**

The risk of consuming herbs and products containing high level of toxins is a considerable threat to patient safety (direct risk). At the same time, the lack of scientific evidence of effect for many complementary therapies and differences in treatment philosophy among complementary and conventional health care providers potentially hinder effective communication about these threats with mutual patients (indirect risk). As such, indirect risk may pose an additional risk to patients who want to combine complementary therapies with conventional treatment in cancer care. Health care providers who care for cancer patients should be aware of these risks.

**Electronic supplementary material:**

The online version of this article (doi:10.1186/s12906-016-1326-3) contains supplementary material, which is available to authorized users.

## Background

Complementary therapies comprise a diverse set of healing philosophies, therapies and products [1], that is generally not taught in conventional medical schools [2]. The definition of complementary provider varies between countries and organizations. We understand complementary providers as “providers other than authorized health personnel who give health-related treatment outside the established health services.” This definition is in line with the Norwegian law on alternative treatment [3]. For example, if an acupuncturist offers acupuncture at a private clinic she or he is a complementary provider. If a physiotherapist practices acupuncture inside a hospital, the treatment is defined as complementary treatment delivered as a part of her or his appointment as a physiotherapist [4]. In Norway and in the EU, the regulation of the complementary profession varies widely [5]. This variation increases the risk and thereby threatens patient safety. However, to become a member of a complementary practitioner organization, a minimum of training in conventional medicine is usually required [6]. It is safe to assume that complementary providers’ knowledge of conventional medicine varies from no formal medical education to being fully trained physicians who have added some complementary modalities to their treatment repertoire.

According to the 2007 National Health Interview Survey (NHIS), which included a comprehensive survey on the use of complementary modalities by Americans, 65% of the respondents who had ever been diagnosed with cancer had used complementary therapies [7]. The prevalence of complementary therapy use is higher among women, individuals with higher education, and those with poorer self-reported health [8, 9].

### Communication

Effective communication among health care providers is essential to providing coordinate care [10]. Optimal communication between health care providers is characterized by the accurate gathering and sharing of information that will be useful to other health care providers, indicating what the patient has been told, planning who will take ongoing responsibility for the patient and keeping the door open for further communication [11, 12]. Effective communication may also contribute to more confidence in health care personnel by the patients and increased likelihood that patients will follow evidence-based recommendations and thereby avoidance of negative interactions between conventional and complementary treatments [13].

The lack of effective communication between patients and health care providers may result in loss of trust within the therapeutic relationship [14]. Furthermore, patients may select harmful, ineffective or expensive complementary modalities, when more effective and less expensive complementary modalities may exist [14]. Communication in clinical encounters experienced by patients as negative may also diminish patient autonomy and self-efficacy and interfere with the self-healing response [15].

Patient-centered communication is the set of skills and behaviors used by health care providers to promote a relationship in which patients actively participate as partners in healthcare decision making and management [16]. A patient-centered relationship involves the concept of mutuality, including power sharing and collaboration between health care providers and patients. Another commonly described element is a “whole person” approach, in which the provider attends not only to the patients’ biological needs, but also to the psychological, social and behavioural dimension of health and illness [17]. A recent Norwegian study among complementary users living with cancer suggests that negative communication experiences in consultations with conventional health care providers may result in the decision to use complementary therapy and even to delay or decline conventional cancer treatment. On the contrary, positive communication experiences lead to the decision to use complementary therapy as a supplement rather than an alternative to conventional medicine [18]. Effective patient-provider communication may be essential in developing patient satisfaction, compliance and positive health outcomes.

Users of complementary therapies report a significantly “lower level of confidence in the efficacy of conventional medicine” than non-users [19]. Nevertheless, most of these patients do not leave the conventional health care system [20] although they may have negative experiences with conventional care. It has been claimed in studies on the quality of health care [21] that when patients are treated with respect, given time to present their health issues, being listened to and given the opportunity to actively participate in the decision-making, they will stay with their general practitioner. A recent Norwegian study revealed that the probability of visiting a complementary provider was lower (12.5 %) among those with a general practitioner relationship lasting more than 2 years compared to those with a shorter relationship (15.5 %) [21].

### Risk

Ideally, patients should be fully informed about the nature of the specific complementary therapies, their potential risks and benefits, and they should have realistic expectations when combining complementary and conventional treatment in cancer care. However, possible risks associated with complementary therapies have been poorly investigated, often due to the assumption that many complementary modalities are considered to be “natural” and, therefore, associated with low risk. Risk associated with any health care is generally separated into direct and indirect risk [4, 22]. *Direct risk* is caused by the treatment itself and is linked directly to the intervention. This dimension includes traditional adverse effects from a treatment, such as bleeding in response to acupuncture needling, or the adverse effects of a herb, as well as risk connected to health advice from the complementary provider. *Indirect risk* is related to adverse effect of the treatment context, e.g., the complementary provider, rather than the intervention. A patient may be harmed by a care context which prevents the patient from receiving the best possible treatment relevant to her or his health needs, e.g., when patients seek a complementary provider for their health complaints which may be effectively treated by conventional medicine (e.g., cancer), and the complementary provider, often unwittingly, causes a delay of conventional treatment [22, 23].

Studies have been conducted to map the communication gap between cancer patients and different health care providers (conventional and complementary) in cancer care [14, 24, 25] However, there is limited research that captures the communication patterns about risk of conventional and complementary health care providers who care for cancer patients. To gain knowledge about this important issue, a literature review with the following aim was performed.

#### Aim

We will examine the qualitative research literature on perception of and communication about the risk of complementary therapies among oncology experts (doctors and nurses), health care physicians and complementary providers and cancer patients. The studies will be summarized into different risk situations.

## Methods

### Searches

The focused question under investigation was:

### How do health care providers (conventional and complementary) perceive and communicate risk of complementary therapies to patients who combine conventional and complementary therapies in cancer care?

The Population Exposure Outcome (PEO) format (tools to formulate questions about qualitative research) was used when searching for relevant articles, which included the following three parts:

**P**opulation: Health care providers, oncology doctors, nurses, physicians and complementary providers

**E**xposure: Risk-communication, risk-perception, risk assessment regarding the use of complementary therapies in cancer care

**O**utcome: Improved communication between different health care providers, improved patient-provider communication, enhance patient safety

The inclusion comprised qualitative studies (individual and group interviews, opinion of an expert and literature reviews) investigating communication and perception about risk of complementary therapies among oncology experts (doctors and nurses), health care physicians and complementary providers and cancer patients. However, qualitative studies that had added a quantitative component e.g., a questionnaire in the design (mixed-design) were also included in the analysis. The studies excluded were quantitative studies and evidence based guidelines.

The following electronic databases were searched: AMED, CINAHL, EMBASE, MEDLINE/PubMed and PsycINFO.

#### Search methods

Depending on the database, various combinations of medical subject headings (MeSH) terms and keywords were used. The searches were combined with the Boolean operators AND/OR. Titles, abstracts and keywords were searched. These MeSH terms were used: *complementary therapies; cancer care unit; communication.*

The following keywords were used: *risk-perception; risk-communication; interpersonal communication; decision making/and risk assessment; neoplasm; oncology; cancer care; cancer center/cancer patient/patient care; complementary and alternative medicine; alternative treatment.* The first author, T.S, performed the searches. T.S, A.K and A.S read the articles and extracted the data.

The findings of all relevant studies published in English were synthesized. The searches were limited to the period January 2000 to February 2015, due to the shift in the acceptance of complementary therapies and its inclusion in conventional health care in the past 10-15 years. Reference lists were hand searched, and further searches of relevant authors were undertaken. (The PubMed search string is attached as Additional file 1 at the end of the manuscript.).

### Methodological assessment of the included studies

The methodological quality of the included studies was assessed using 10 questions from the Critical Appraisal Skills Programme (CASP) checklist for qualitative research www.casp-uk.net. This checklist is validated and recommended by The Norwegian Knowledge Centre for Health Services. First, three broad issues were considered: “*Are the results valid?”*, “W*hat are the results?”* and “*Will the results help locally*?” Then two screening questions were considered. If the answer to both was “*yes*”, the studies were assessed according to eight detailed questions (Table 1).

**Table 1:** 10 questions for appraising qualitative research (CASP checklist)

Screening Questions	Responses		
1. Was there a clear statement of the aims of the research?	Yes	Can’t tell	No
2. Is a qualitative methodology appropriate?	Yes	Can’t tell	No
Detailed questions			
3. Was the research design appropriate to address the aims of the research?	Yes	Can’t tell	No
4. Was the recruitment strategy appropriate to the aims of the research?	Yes	Can’t tell	No
5. Was the data collected in a way that addressed the research issue?	Yes	Can’t tell	No
6. Has the relationship between research and participants been adequately considered?	Yes	Can’t tell	No
7. Have ethical issues been taken into consideration?	Yes	Can’t tell	No
8. Was the data analysis sufficiently rigorous?	Yes	Cant’ tell	No
9. Is there a clear statement of findings?	Yes	Cant’ tell	No
10. How valuable is the research?	Yes	Cant ‘tell	No

^a^ www.casp-uk.net

### Design

We included qualitative studies in this review because most of the so far limited knowledge on risk perception and risk communication in clinical settings involving cancer patients’ use of complementary therapies is based on explorative, qualitative studies. A qualitative methodological approach can give access to in-depth information on the perspectives and practices of various stakeholders involved in clinical settings. This includes information on experiences from different interventions applied in practice [26]. In general, qualitative studies may contribute to deeper understanding and thorough knowledge of different stakeholders’ perspectives on health and well-being in terms of e.g., risk, communication and decision-making, especially in situations in which we have limited previous knowledge of our phenomenon of interest [27].

### Data analysis

We used qualitative content analysis [28] to analyze the results from the included studies. The goal was to provide knowledge and understanding of the phenomena of interest through a systematic classification process of coding and identifying themes [29]. The codes were identified before (“direct and indirect risk situations”) and during the data analysis. Hence, the coding was a mixed type, using elements from conventional and direct content analysis [29]. From this analysis, concepts and themes were developed. At each stage of the analysis process, two researchers (AS and TS) met and discussed after having read the relevant data several times and made individual lists of coding suggestions. Data and information obtained from the included studies will be presented as results in this study. These results do not represent the authors’ view or understanding of complementary therapies or conventional medicine.

## Results

### Searches

**Fig. 1:**
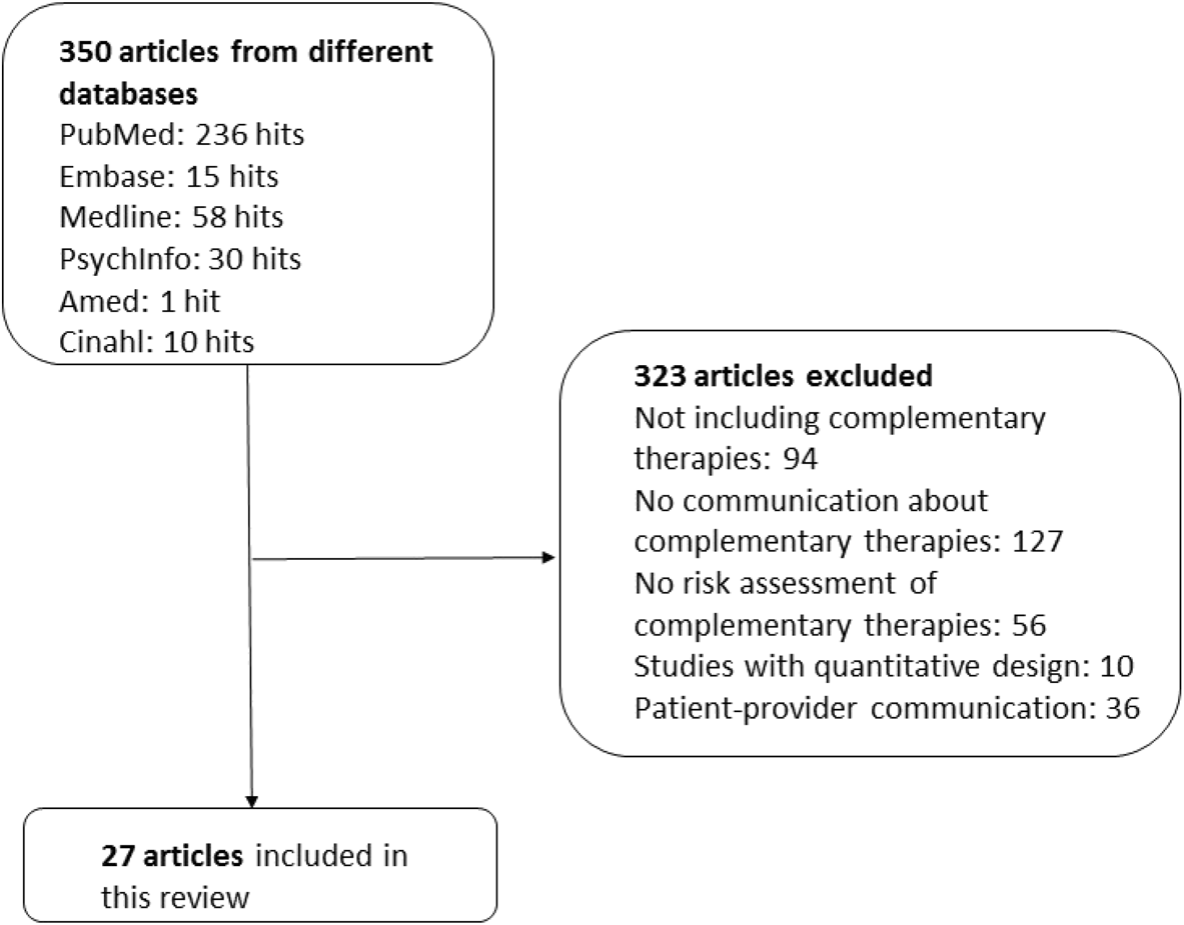
Flow chart of the inclusion process

The literature search resulted in 350 potentially relevant studies. All abstracts were screened for relevance. After excluding 323 irrelevant studies (94 studies not including complementary therapies, 127 studies not including communication about complementary therapies, 56 studies not including risk assessment of complementary therapies, 10 studies had a quantitative design and 36 studies comprised patient-provider communication), a total of 27 studies were included in this literature review (Fig. 1).

Twenty-seven studies [14, 23–25, 30–52] with a variety of qualitative study designs, such as focus groups and individual interviews, videotaped studies, literature reviews, opinion of experts and mixed studies were included in this review. Of these studies, 12 included complementary providers [35–42, 48, 50–52], 5 included oncology experts (doctors and nurses) [24, 25, 43, 44, 46] and 11 included physicians [14, 23, 30–34, 45, 47, 49, 52].

### Main themes

During the analysis, five main themes were revealed: *Risk perception* (based on a risk-benefit comparison), *direct risk situations* (adverse effects, interactions and toxicity of a complementary intervention), *indirect risk situations* (ethical risks, different models of disease causality and treatment philosophy, lack of regulation, standardized education and common medical terminology, harms of treatment regimen), *risk communication* (ineffective patient-provider relationship, cancer patients who delay or decline the use of conventional medicine, how to talk about complementary use (factors that enhance or obstruct communication), and *information regarding complementary therapies and conventional medicine* (lack of knowledge).

### Risk perception based on a risk/benefit comparison

Seven papers addressed this theme [24, 25, 30–33]. The theme was based on a risk/benefit comparison meaning that the benefits have to clearly outweigh risks connected to the use of a complementary modality for health care providers to consider recommending it to patients. The stronger the evidence for safety and efficacy, the stronger the argument that the modality should be recommended [24, 32]. However, health care physicians and oncology experts find it difficult to recommend a complementary modality because they doubt that these modalities are beneficial due to limited scientific evidence [14, 33]. The oncologists are particularly critical of complementary therapies due to lack of scientific evidence [24]. Moreover, physicians perceive complementary therapies as a backup when conventional medicine has little to offer for the patient. According to these physicians, the reasons for offering complementary therapies included being able to offer patients something that would “do no harm” as an addition to pharmacological drugs that may have unpleasant adverse effects [32]. It is, therefore, important to distinguish between complementary modalities that give false hope of cure and those that offer supportive care to improve the patients‘sense of wellbeing [24, 25, 32].

### Direct risk situations

Direct risks were identified as *adverse effects, interactions and toxicity* of complementary therapies in this review. We found that eight studies addressed this theme [24, 25, 30, 35–37, 49, 51]. Patients should be fully informed of the nature of the specific complementary modalities, potential risks and benefits, and realistic expectations. Complementary products that may interact with conventional cancer treatments, should not be used concurrently with chemotherapy, as they lessen the effectiveness of the latter [24, 25, 35]. However, it is possible to use other complementary products (that do not interact) concurrently with anticancer drugs [35]. This information should be communicated to cancer patients so that they can take this into consideration in their decision-making. The directive 2001/83/EC of the European Parliament and of the Council of 6^th^ November, 2001 on the Community code relating to medicinal products for human use states that “no medicinal product may be placed on the market of a member state unless a marketing authorization has been issued by the competent authorities of that member state in accordance with this delivery or an authorization has been granted in accordance with Regulation (EEC) No 2309/93” [53]. Outside the EU many products are not undergoing appropriate quality control, and precautions should be taken in many cases. According to information obtained from studies included in this review, Ayurvedic medicines from India and herbs and products from the Middle East may have high heavy metal content, and may contain bacteria and toxic organic substances [30, 37, 51].

### Indirect risk situations

Twenty-two studies addressed indirect risk situations [14, 24, 25, 31–42, 44, 45, 47, 49–52]. Health care physicians and oncology experts have an *ethical responsibility* to initiate the communication regarding the use of complementary therapies with cancer patients [45]. However, according to data obtained from this literature review, oncology doctors and physicians will discuss complementary therapies only when a patient him/herself raises this issue within a consultation. This passive attitude was linked to a lack of sufficient scientific evidence for positive outcomes of complementary therapies found in high quality randomized controlled trials (RCTs) [32]. Oncology nurses, on the other hand, sometimes actively promote complementary modalities that they find to correspond with their vision of holistic care [44].

According to the included studies, complementary providers often differ from conventional health care providers in their understanding of treatment concepts, philosophies and diagnostic procedures. This leads to different *models of disease causality* (cells, blood, nerves vs. energy, vital force, meridians) and *treatment philosophy* (reductionism vs. holism). As many complementary providers are philosophically oriented towards personal and spiritual growth, patients may feel guilty if the disease continues to advance despite the patients’ best spiritual and mental efforts [38]. According to Broom and colleagues [24, 30], such philosophies may also give patients false hope of recovery.

Another indirect risk connected to the combination of conventional and complementary treatment in cancer care is the lack of regulation and standardized education in many countries. Currently, there are, for example, no standard training requirements for complementary providers working in cancer care or any other health care setting [42] in the EU [5]. According to Mackareth et al., complementary providers in England need specific training to learn how to practice safely [54].

Moreover, there is a need for *common medical terminology* to bridge the communication gap between health care providers working outside the conventional health care system [31, 37]. Common medical terminology may reduce the existing communication gap between conventional and complementary providers about mutual patients. To minimize communication gap between physicians, oncology experts and complementary providers, a medical complementary record should include a treatment plan with conventional and complementary diagnosis, explanation of terminology, possible treatment interactions, description of the complementary treatment plan and goals. If possible, the quality of any complementary supplement given should be reported [52].

### Risk communication

A total of 18 studies addressed the theme of risk communication [14, 23–25, 30–33, 35–38, 43, 46–48, 51, 52]. Well-functioning communication has been associated with trust in conventional health care and positive health outcomes. An open and equal dialogue in clinical practice may decrease risks associated with malpractice, maximize positive treatment outcomes and avoid possible adverse effects that may occur due to combinations of complementary and conventional cancer treatment [14]. However, research has revealed that oncology experts and their patients often have significantly different perspectives on the legitimacy of complementary therapies and the understanding of cancer in a broader sense [24]. According to data obtained from this review, medical knowledge and patients’ perceptions of risks and benefits associated with complementary and conventional cancer care are incompatible when cancer patients choose to *delay or decline conventional treatment* [23]. These patients challenge the rationality of medical advice and the authority of the oncology experts. On the other hand, conventional health care providers express that they want patients to actively participate in the decision-making, but in line with their own views of what constitutes an effective complementary modality [32]. According to Ben-Arye and colleagues cancer patients should be asked about their use of complementary modalities. This will strengthen *the patient-doctor relationship* [34] and patients favor complementary providers who are regulated health professionals [42]. Contrariwise, complementary providers perceive themselves as gatekeepers for the patients. They mediate between patients and physicians and serve as a bridge between patients and their families and the oncology experts. In this sense, complementary providers contribute to improved patient-physicians dialogues [48].

To enhance *communication with patients,* it is better for health care physicians to admit uncertainty regarding the benefits and safety of a complementary modality. Addressing uncertainty may have a protective value by allowing for space and hope [14]. However, as mentioned above, many health care physicians will only discuss complementary therapies when a patient raises the issue in a consultation because the scientific evidence for complementary therapies is weak. Moreover, health care physicians endorse complementary modalities that are the closest to conventional medicine [32]. Oncology nurses, on the other hand, are the patients’ advocate [24] and often mediate between oncologists and patients [44].

Many oncologists perceive that time spent visiting complementary providers is of little benefit for patients who are in a palliative stage of cancer [35]. Moreover, many oncologists are skeptical to many complementary treatments because they are expensive, and patients mostly have to pay for them out of pocket. In this sense, both financial costs and time loss are perceived as *communication barriers* [25, 30, 35].

### Information regarding complementary therapies and conventional medicine

Nine papers addressed the theme “information regarding complementary therapies and conventional medicine” [24, 30–32, 42–44, 46, 50]. *Lack of knowledge about complementary therapies* may represent a threat to well-functioning communication. Data from this review demonstrates that there is lack of knowledge regarding the use of complementary therapies and complementary products among oncologist experts (doctors and nurses) and health care physicians. According to Kemper and colleagues an Internet education program about safety of complementary therapies and dietary supplements to improve knowledge, confidence and communication practice among health care providers in a health care setting, reported significant and sustained improvements in knowledge, confidence and communication practices [55]. On the other hand, conventional health care providers do not have to be experts in complementary therapies to have a respectful, balanced and helpful discussion about complementary therapy use with patients [25].

A number of naturopathic medical schools in the US are accredited by the US Department of Education, and these providers have high levels of medical knowledge. To treat patients, complementary providers must have knowledge of the biomedical concepts of pathology [31]. However, due to lack of regulation and standardized education in many countries, complementary providers may lack adequate medical training. Research shows that complementary providers want to be involved in cancer care and are enthusiastic about a professional education program [42, 50]. For example, they may have limited knowledge regarding interactions of herbal supplements and anticancer diets with conventional treatment, showing a need to regulate complementary training and professions [35] (Table 2).

**Table 2:** Description and methodological assessment of the included studies

Study ID	Objectives	Participants	Setting	Research design	Methodological assessment (are the results of the study valid? Yes/No/Can’t tell)	Main findings (themes)	Country	Funding
Barrett B, 2000 [38]	The nature of practice, healing philosophy, choices of therapeutic methods and ideas about the use of therapeutic modalities	17 CAM and conventional medicine patients and 20 CAM practitioners	Home office, public places in the Madison, Wisconsin area	Semi structured interviews	Yes	Risk communication and perception, indirect risk and knowledge about CAM	USA	Not reported
Baynham-Fletcher L, 2008 [39]	Standard for credentialing CIM^a^ therapies between-state and between-institutions	Credentialing process	MD Andersen cancer setting	Descriptive study of the credentialing process for CIM^a^ practitioners	Can’t tell	Information, knowledge and indirect risk about CIM^a^	USA	Not reported
Ben-Arye E, 2012 a [34]	Patient-provider communication about complementary approaches and implementation and integration of CM^b^ in health care	23 articles	Spescial issues of the Journal Patient Education and Counseling	Assessing articles of different qualitative methods, such as literature reviews, empirical descriptive studies and interviews	Can’t tell	Risk communication and indirect risk	International	Not reported
Ben-Arye E, 2012 b [51]	CAM research in support cancer care	85 articles in Arabic, Hebrew, French and Turkish	Medline/PubMed, ULAKBIM the Turkish Academic network and Information Centre	Literature review	Yes	Risk communication, direct and indirect risk	Israel, Turkey, Iran, Saudi-Arabia, Palestine, Jordan and Egypt	Not reported
Ben-Arye E, 2013 [40]	Provide oncologists with models for Integrative CM^b^ within supporting care	Physicians leading six integrative oncology practices	MD Andersen, Penny Brohn, Herdecke Community Hospital, Lin Medical Center, Rambam Health Care Campus, Fundaleu Institute	Descriptive analysis of key elements which facilitates CM^b^ integration	Yes	Indirect risk and communication	USA, UK, Germany, Israel and Argentina	The Israeli Society for Complementary Medicine, the UK College of Medicine, Bnai Zion Medical Center, the Technion-Israel Institute of Technology, Lin Medical center of Clait Health Servises, the Academic Study Group for Israel and the Middle East
Broom A, 2009 [24]	How oncologists and oncology nurses engage and communicate risks with patients about CAM	13 oncologists, 12 oncology nurses	Two main hospitals in a state capital city	In-depth interviews	Yes	Risk perception, direct and indirect risk, risk communication, information about CAM and CM^e^	Australia	Not reported
Broom AF, 2013 [30]	Excamine oncology clinicians’ accounts of communication with their cancer patiens	16 medical specialists, 5 oncology nurses and 1 oncology clinical psycologist	Three hospitals and one palliative care service in Dehli	In-depth interviews	Yes	Direct risk, indirect risk and risk communication, information of CAM and CM^e^	India	Australian Research Council (FT100100294)
Fox P, 2012 [35]	Different perspectives regarding the role of CAM in the cancer setting	31 women with breast cancer, 13 oncology nurses, 7 oncologists and 20 CAM practitioners	Not reported	Semi structured interviews	Yes	Indirect risk, direct risk and risk communication	Ireland	Not reported
Fox P, 2013 [36]	Rate and type of CAM used by women with breast cancer, reasons and perceptions of the utility of the CAM terapies used	20 oncology professionals (13 oncology nurser, 7 oncologists), 20 CAM practitioners	Not reported	Semi structured interviews, survey	Yes	Indirect risk, direct risk, risk communication	Ireland	Irish Cancer Society
Frenkel M, 2010 [14]	Overview of the literature regarding communication in cancer care related to the use of CAM. Discuss a possible model of effective patient-physician communication		Not reported	Literature overview	Can’t tell	Indirect risk, risk perception, risk communication	International	No financial support
Goldstein, 2003 [41]	The role of CAM in oncology			Theoretical paper	Can’t tell	Indirect risk	USA	Not reported
Jason SL, 2009 [46]	Examine CAM discussion of oncologists, patients and companions on first time consultation visits at comprehensive outpatient clinic for CAM discussions	93 video tape consultation interactions of 13 oncologists, 93 patients and 82 visit companions	National Cancer Institute-designated cancer senter and teaching hospital outpatient clinic in a large urban Midwestern city	Qualitative observational study	Yes	Risk communication, information about CAM and CM^e^	USA	Not reported
Klimenko E, 2007 [31]	Investigate definition of health, disease and healing	4 medical physicians, 2 psychiatrists, 1 psychologist and 7 CAM practitioners		Delhi process	Yes	Risk perception, indirect risk, risk communication, information about CAM and CM^e^	USA	Not reported
Mackereth PA, 2009 [42]	Uncover complemetary therapists’ motivation to work in cancer/supportive and palliative care setting	19 nurses, 2 doctors, 3physiotherapists, 27 CAM therapists	One hospice, one cancer care hospital, one cancer care senter	Questionnaire survey followed by semi-structured interviews	Yes	Indirect risk, information about CAM and CM^e^	UK	The Big Lottery Fund
Mackereth PA, 2010 [50]	Investigate CAM providers’ challenges of working in cancer care settings and the value of clinical supervision	15 CAM therapists	Five cancer care senters	Focus group interview	Yes	Indirect risk	UK	Not reported
Madjar I, 2007 [47]	Provide insigth into how physicians perceive cancer patients who decide to forgo or stop medically recommended therapies	12 medical and radiation oncologists	Two regional oncology senters	Individual semi-structured interview	Yes	Risk communication, indirect risk	Israel, Australia	Israeli Cancer Association, Newcastle Mater Hospital Margareth Mitchell Research Fund, Australia
Maha N, 2007 [32]	Explore academic doctors’ use of CAM and its role within the NHS^c^, along with rationales given for these views	9 doctors with a dual clinical and academic role	Study participants’ work place	Semi-structured interview	Yes	Indirect risk, risk perception, risk communication, information about CAM and CM^e^	UK	Not reported
Mazor KM, 2013 [43]	Assess patient-centered communication in cancer care, stakeholder perspective	37 cancer patients, 17 family members, 52 clinicians	Cancer research networks, cancer communication research senter	Semi-structured interview	Yes	Indirect risk, risk communication, information about CAM and CM^e^		A grant from Cancer Communication Research Center (P20CA137219). Cancer research network pilot grant (U19CA79689), grant from National Center for advancing Translational Science ((UI1TR000161)
Popper-Giveon A, 2012 [48]	Assess the role of CAM therapists who treat cancer patients to promote patients’ well-being during chemotherapy and advance stage of disease	27 Arabic therapists (folk healers, complementary therapists and religious-spiritual healers)	Not reported	Semi-structured interviews	Yes	Risk communication	Israel	A grant from Clalit Research Institute by Clalit Health Services
Popper-Giveon A, 2013 [37]	The attitudes towards integrative medicine among CTM^d^ terapists’ who treat Arab cancerpatients in Israel	27 arab therapists (folk healers, complementary therapist and religious-spiritual healer)	Not reported	Semi-structured interviews	Yes	Indirect risk, direct risk, risk communication	Israel	A grant from Clalit Research Institute by Clalit Health Services
Richardson P, 2012 [44]	Highlight the importance of spirituality and religion in cancer care		Not reported	Literature review	Can’t tell	Indirect risk, information about CAM and CM^e^	USA	Not reported
Roberts D, 2005 [33]	Discuss the current policies, perceptions and expectations regarding the use of complementary therapies in cancer care	Different policy documents		Literature review	Can’t tell	Indirect risk, risk communication, risk perception	UK	Not reported
Salamonsen A, 2015 [23]	Doctor-patient communication and how this communication influences treatment decisions	9 patients from the Registry of Exceptional Courses of Disease	Participants’ home or other places where the participants were comfortable	In-depth interview	Yes	Risk communication	Norway	No financial support
Schiff E, 2011 [52]	Improve communication between physicians and CAM practitioners	One main panel (*n* = 16), one extended panel (*n* = 25), one conference discussion forum (*n* = 247), survey (*n* = 1254)	Various places	Delphi process and one survey	Yes	Indirect risk, risk communication	Israel	Not reported
Schofield P, 2010 [25]	Discuss CAM in oncology consultations and develop quidelines	37 included papers	MedLine, Cinahl and PsyInfo	Systematic literature review	Yes	Risk perception, direct risk, indirect risk, risk communication	Australia	The National Breast and Ovarian Cancer Centre
Verhoef M, 2007 [49]	Evaluate a research framework for cancer care using a complex whole system research model			Methodological paper/whole system research	Yes			
Verhoef MJ, 2008 [45]	Physicians’ responsibility to discuss CAM with patients. Ethical perspectives			Opinion of an expert	Yes	Indirect risk	Canada	Supported by The Canadian Cancer Society

^a^CIM: Complementary and Integrative Medicine, ^b^CM: Complementary Medicine, ^c^NHS: National Health Service, ^d^ CTM: Complementary and Traditional Medicine, ^e^Conventional Medicine

### Methodological assessment of the included studies

All studies reported a clear statement of the objectives and described the study participants. The research design comprised 11 semi-structured interviews, 2 descriptive studies, 5 literature reviews, 2 Delphi processes, 1 focus group interview, 2 methodological/theoretical papers, 1 opinion of an expert, 1 qualitative observational study (video tape), 2 mixed method studies (survey and interview (*n* = 1) and one study included both a Delphi process and a survey (*n* = 1). The studies were methodologically evaluated according to the CASP checklist. The majority of the studies (*n* = 21) had valid results, and for six studies (*n* = 6) the validity of the results could not be ascertained. Four of these studies (*n* = 4) were literature reviews that did not report the methodology of the literature searches [14, 33, 34, 44]. One theoretical paper did not provide a rationale for the references included [41] and one study provided inadequate description of the credentialing process [39]. Based on this evaluation we concluded that the methodological quality of the majority of these studies was generally high (see Table 2).

## Discussion

A wealth of qualitative evidence was revealed in this systematic review of the literature. The qualitative evidence was mainly based on individual interviews, focus group interviews and literature reviews. Two-thirds of the studies provided valid results, while one-third provided less certain results, according to the CASP checklist. As such, these studies represent the most comprehensive set of risk perception and communication to date among different health providers (conventional and complementary) involving patients’ use of complementary therapies in cancer care.

### Direct risk situations

Some complementary products may be contaminated or contain high levels of toxins. This is an important risk since consuming herbs with, for example, high doses of mercury or lead, may be risky for patients [40, 56, 57]. Ayurvedic medicines, for example, are divided into 2 major types: herbal only and *rasa shastra* [57]. Rasa shastra is an ancient practice of deliberately combining herbs with metals, such as mercury, lead, iron and zinc, or minerals, such as mica and gems (e.g., pearls). Rasa Shastra practitioners claim that these medicines are safe and therapeutic, if properly prepared and administered (including processes of purification, to remove undesirable qualities and enhance therapeutic power). However, in a random sample of commercially prepared Ayurvedic medicines purchased on the Internet, nearly 21 % contained detectable levels of lead, mercury or arsenic [57]. Thus, daily dose limits of toxic metals and quality tests of these products are warranted to enhance patient safety.

### Indirect risk

Risk connected to clinical practice (indirect risk) was another threat to patient safety. It is primarily the difference in disease causality and treatment philosophy that seems to impede effective communication between conventional and complementary providers. These differences in epistemology and philosophical values may also influence a feasible collaboration across professions negatively.

### Risk communication

Moreover, the non-communication between conventional and complementary health care providers is risky for patients who have to integrate what they perceive to be the best of conventional and complementary treatment themselves. Negative experiences from doctor–patient interaction and outcomes of conventional treatment can influence the cancer patients’ decision to use complementary therapies, and to potentially decline or delay conventional treatment [18]. Patients’ negative attitudes toward conventional treatment have also been linked to possible adverse effects of treatments [24, 58].

### Risk perception

Conventional health care providers find it difficult to recommend a complementary modality with low or no scientific evidence of effect. The complementary providers, on the other hand, claim that the methods used to investigate effect (RCT research) are not suitable for complementary therapies, since complementary therapies treat the “whole person” and not a disease as such [59]. However, researchers may investigate one disease, but single agents are rarely applied as a treatment intervention, which makes it difficult to apply RCT designs. Studies of patients’ perspectives on benefits and risks associated with the use of complementary therapies have revealed that these patients experience other benefits from complementary modalities, which may be missed in some of the outcomes traditionally utilized in RCT research [60, 61]. Moreover, cancer patients who want to combine complementary and conventional treatment differ from oncology experts in their perceptions of risk associated with the use of both complementary and conventional cancer treatment [23].

Discussions and analyses of issues regarding safety and risk of complementary therapies primarily involve issues of direct risk, usually including adverse effects and the monitoring of these effects (e.g., surveillance). However, direct risk forms only a part of the risk profile of any medical treatment. Findings from this literature review indicate that risk associated with clinical practice (indirect risk) may pose an additional danger to patients. This is in accordance with Wardle and Adams [62] who claim that indirect risk in complementary therapy use is generally similar to that associated with conventional medical care. However, in the complementary field these risks may be more severe due to lack of appropriate regulation and legislation. In a study about complementary therapy regulations in Europe, Wiesener and colleagues [5] found that “what was defined and regulated as complementary therapies in one country, could be regulated either as conventional, complementary or alternative medicine in other European countries, or not regulated at all”. In some countries, for example, one must be a medical doctor to practice homeopathy, but in a neighboring country one may practice homeopathy if registered as a “natural health practitioner”. These researchers found that CAM in Europe is not regulated according to any current theories dealing with risk and patient safety. Consequently, CAM users, health providers, researchers, and authorities have insufficient information about the risk and safety situation, and every stakeholder interprets the situation differently. This situation in itself influences patient safety and risk understanding [5, 63].

### Methodological limitations

Strong efforts have been made to retrieve all studies on the subject, but one cannot be absolutely certain that all existing studies have been identified. However, a medical librarian with extensive research expertise supervised the first author when she performed the searches and controlled the searches for bias. This methodological approach may have minimized the possibility for selection bias in this ﻿present review.

### Practical implications

To provide safe and cooperative care, better cross education is needed among health care providers with and without complementary training. Complementary providers should have a solid and updated education in conventional medicine and their complementary-specific modality, and conventional health providers should be willing to seek a minimum of information regarding complementary therapies. This step may improve the current communication gap between different health care providers and their patients. As mentioned, therapies out of the spectrum of complementary therapies are generally not licensed and are equally unregulated. Moreover, many complementary modalities are derived from traditional systems of medicine [64] with limited or no research tradition. Therefore, there is little knowledge about efficacy, effectiveness and biological mechanisms. Many products are not subject to regulation, and some may be toxic. These issues may also be key drivers in the communication challenges between different health providers.

### Implication for further research

There is a lack of research that investigates how complementary providers communicate with their patients about the risks of combining complementary and conventional treatment in cancer care. Complementary providers’ attitudes towards conventional cancer treatment and how these attitudes are communicated to cancer patients have also not been adequately investigated. Research that focuses on how complementary providers’ perceptions about conventional cancer treatment influence patients’ treatment choices is needed as well.

## Conclusion

Direct risk such as the risk of consuming herbs and products containing high level of toxins is a considerable threat to patient safety. Indirect risks such as the lack of scientific evidence of effect for many complementary therapies and differences in treatment concepts and philosophy may hinder effective communication between different health care providers in cancer care. As such, indirect risk may pose an additional risk to cancer patients who want to combine complementary therapies with conventional treatment. Health care providers who care for cancer patients should be aware of these risks.

## Additional file

Additional file 1: The PubMed search string. (DOCX 46 kb)

## Abbreviations

CIM: Complementary and integrative medicine
CM: Complementary medicine
CTM: Complementary and traditional medicine
NHS: National Health Service
