# Supplementary figures and images for: Perception of risk and communication among conventional and complementary health care providers involving cancer patients’ use of complementary therapies: a literature review
Source: BMC Complement Altern Med. 2016 Sep 8;16(1):353. doi: 10.1186/s12906-016-1326-3 (PMC5016861; doi:10.1186/s12906-016-1326-3)

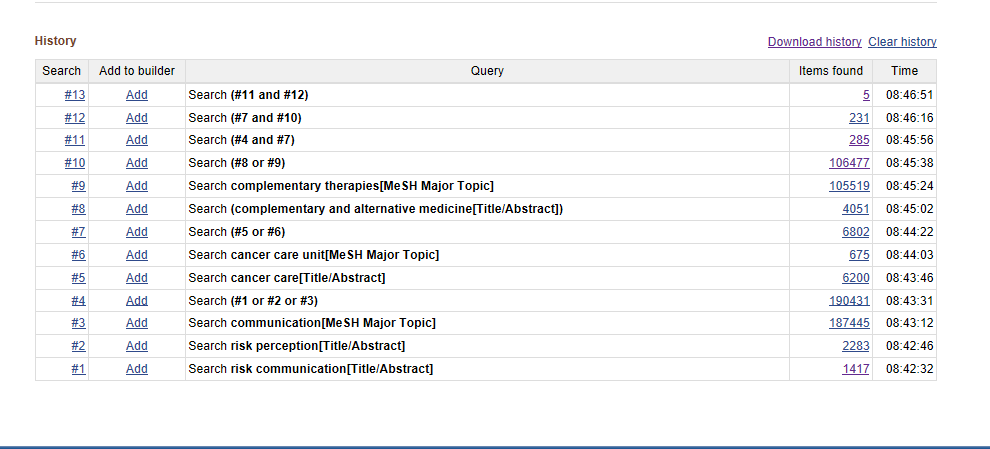

Supplement: Additional file 1: — The PubMed search string. (DOCX 46 kb) [file 12906_2016_1326_MOESM1_ESM.docx]
